# Bayesian profiling of molecular signatures to predict event times

**DOI:** 10.1186/1742-4682-4-3

**Published:** 2007-01-19

**Authors:** Dabao Zhang, Min Zhang

**Affiliations:** 1 Department of Statistics, Purdue University, 150 N. University Street, West Lafayette, Indiana 47907-2067, USA

## Abstract

**Background:**

It is of particular interest to identify cancer-specific molecular signatures for early diagnosis, monitoring effects of treatment and predicting patient survival time. Molecular information about patients is usually generated from high throughput technologies such as microarray and mass spectrometry. Statistically, we are challenged by the large number of candidates but only a small number of patients in the study, and the right-censored clinical data further complicate the analysis.

**Results:**

We present a two-stage procedure to profile molecular signatures for survival outcomes. Firstly, we group closely-related molecular features into linkage clusters, each portraying either similar or opposite functions and playing similar roles in prognosis; secondly, a Bayesian approach is developed to rank the centroids of these linkage clusters and provide a list of the main molecular features closely related to the outcome of interest. A simulation study showed the superior performance of our approach. When it was applied to data on diffuse large B-cell lymphoma (DLBCL), we were able to identify some new candidate signatures for disease prognosis.

**Conclusion:**

This multivariate approach provides researchers with a more reliable list of molecular features profiled in terms of their prognostic relationship to the event times, and generates dependable information for subsequent identification of prognostic molecular signatures through either biological procedures or further data analysis.

## Background

High-throughput biotechnologies such as microarray and mass spectrometry permit simultaneous measurements of enormous bodies of genomic, proteomic, and metabolic information to be made. Such information helps us understand the molecular basis of important clinical outcomes, and thus improves the efficiency as well as accuracy in clinical decision making. More specifically, a small subset of these molecules can be used as biomarkers in daily clinical practice for detecting disease at early stages, measuring disease progress, monitoring the efficacy of treatments, and potentially accelerating the drug discovery process. However, the promise of genomics, proteomics, and metabolomics in clinical medicine rests on identifying these disease-specific molecular signatures. Clinical and preclinical studies of patients' genomics and proteomics profiles usually present datasets that share common characteristics, i.e., many molecular features ("large *p*") collected from few individuals ("small *n*"). The statistical challenge is to mine prognostic signatures from thousands of candidates by efficiently extracting information from samples of limited size, i.e., "small *n *large *p*" datasets. Moreover, the clinical outcomes measured for certain patients, e.g., survival times of cancer patients, are usually censored data, which further complicates the statistical analysis. There has been extensive research on the classification and prediction of cancer using gene expression information [1-3], but there has been less progress in identifying individual molecules that can be used to predict the clinical outcome. We devote this paper to developing a Bayesian approach to profile molecular features on the basis of their prognostic relations to event times.

The proportional hazard model [4] has a long history in modeling the association of risk factors to the right-censored event times observed in clinical study [5,6]. Through this model, it has been of special interest to develop a systematic approach to identifying molecular signatures for event times with "small n large p" datasets. However, the overwhelmingly larger number of molecular candidates compared to the number of individuals prohibits exhaustive variable selection because of the heavy computation and model-overfitting considerations. A variety of strategies have been proposed in the literature. The first is to reduce the list of genotypic candidates by univariately associating each of them with phenotypic clinical outcome [1,7], and then regress the clinical outcome on the selected candidates. The second employs principal component analysis (PCA) to build up "eigengenes" (i.e., linear combinations of genes) and associates these with phenotypic clinical outcomes, and the identification of molecular signatures is further explored on the basis of these [8]. The third strategy employs partial least squares (PLS) [9,10] to construct orthogonal "eigengenes" [11]. Other strategies have also been used to reveal interesting prognostic molecular signatures for certain event times [12-15]. Recently, Tadesse et al. [16] proposed a Bayesian error-in-variable survival model to identify genes of which the expression levels are associated with survival outcome. It is widely accepted that most genes measured in microarray experiments provide little information for predicting patient survival, so a necessary step in the analysis is to reduce the number of candidates before identifying prognostic molecular signatures with a relatively small sample. This reduction is usually carried out by ranking molecular features (either the original molecular candidates or the "eigengenes") according to either z scores [7] or Cox scores [17-19], which measure the univariate association of each molecular feature with the event time. Several top-ranked molecular features are further explored for their prognostic associations with the event time. As shown in our simulation study, employing the univariate Cox scores to profile molecular features can be misleading as it may miss many important candidates but select many false-prognostic ones. Indeed, molecular features with high univariate association to the event time may not necessarily predict the event time effectively when applied together. As shown by Sha et al. [20] and Tadesse et al. [21], the disease may often be affected jointly by subsets of the genes while each individual gene might have a relatively weak effect. This study focuses on developing an efficient yet robust approach to profiling molecular features on the basis of their prognostic associations with the event time, taking advantage of the Bayesian framework for the proportional hazard model proposed by Kalbfleisch [22].

We acknowledge the high correlation between some molecular features due to the complicated genetic architecture. For example, genes involved in the same metabolic pathway may be similarly or oppositely regulated. These closely-related molecular features can result in collinearity between the candidates, and should therefore be grouped together in order to address their prognostic associations with the event time properly. Here, we group closely-related molecular features into linkage clusters. A centroid "gene" is constructed to represent each linkage cluster and thus partially solve the collinearity issue. As univariate Cox scores are unable to account for the complicated correlation structures among molecular features, we employ the Bayesian approach to construct a natural framework for molecular feature profiling.

We first propose a two-stage procedure for profiling prognostic molecular signatures for event times, and present the construction of linkage clusters as well as their centroids. A Bayesian framework of the Cox proportional hazard model is specified for "large *p *small *n*" data and a profiling criterion is described accordingly. The performance of our approach is evaluated via a simulation study and application to data concerning diffuse large B-cell lymphoma (DLBCL) [15].

## Results

### Simulation study

**Figure 1:**
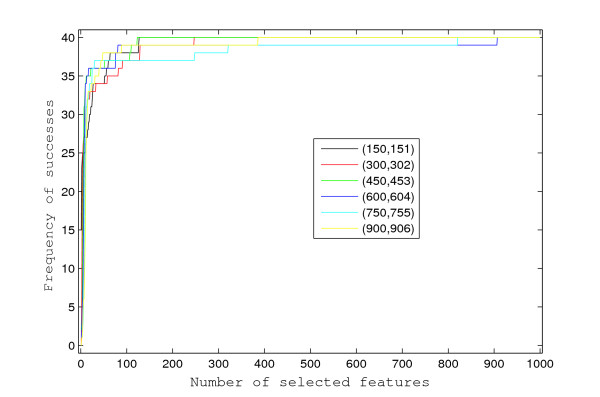
**Frequency of successes using the Bayesian approach**. For each of the six feature pairs, the frequency of successes (*y*-axis) is calculated as the total number of correct detections in the 20 simulated datasets when the Bayesian approach is used to select a certain number of features (*x*-axis).

**Figure 2:**
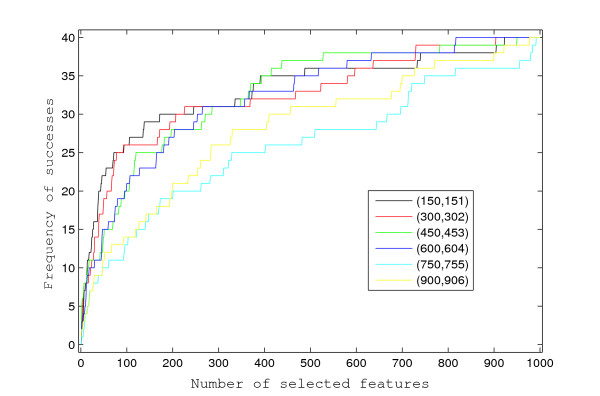
**Frequency of successes using univariate Cox scores**. For each of the six feature pairs, the frequency of successes (*y*-axis) is calculated as the total number of correct detections in the 20 simulated datasets when univariate Cox scores are used to select a certain number of features (*x*-axis).

**Figure 3:**
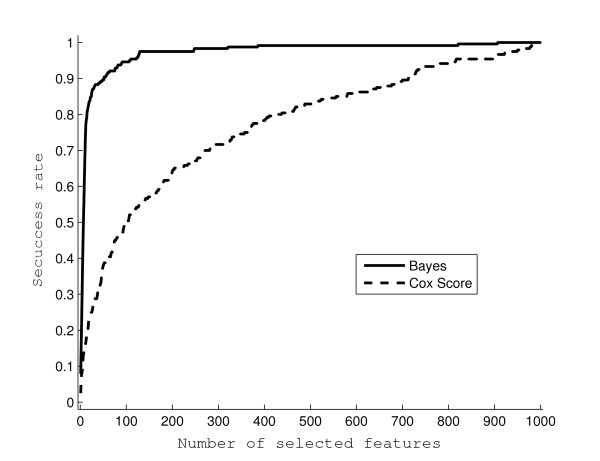
**Comparison between the Bayesian approach and Cox scores**. Shown as success rates (*y*-axis) are the true positive rates when a certain number of features (*x*-axis) are selected in each of the 20 simulated datasets. The solid line represents the results from the Bayesian approach and the dotted line represents the results using univariate Cox scores.

To evaluate the performance of the proposed approach, we simulated 20 survival datasets, each having *p *= 1, 000 features and *n *= 125 independent individuals. The feature values were generated from an autoregressive process of order one, with autocorrelation *ρ *= 0.5 and unit variance white noise. The event times follow an exponential distribution of which the rate is determined by a linear combination of the 12 features with non-zero coefficients. Independent random censoring times were generated from standard exponential distributions, and this induced censoring of approximately 50% of the observed event times. Among the 1, 000 autocorrelated features, the indices of the 12 with non-zero coefficients are 150, 151, 300, 302, 450, 453, 600, 604, 750, 755, 900, 906, and their values alternate between 1 and -1. Such constant-magnitude coefficients were chosen in order to evaluate the effect of correlation among features on the profiling, as the correlations between the pairs of features, i.e., (150, 151), (300, 302), (450, 453), (600, 604), (750, 755), and (900, 906), decrease geometrically from 0.5 to 0.015625. As shown in Figure 1, these feature pairs have similar chances of being selected as top features while being ranked by the Bayesian approach. In this simulation study, the proposed Bayesian approach could select each non-zero coefficient feature with high probability (more than 0.8) when more than 12 features were selected in total. However, when univariate Cox scores are used, a feature pair with higher correlation is more likely to be among the selected top features, and in general, all 12 features are less likely to be correctly selected, as shown in Figure 2. The percentages of the 12 non-zero coefficient features selected into top features (i.e., success rates) are shown in Figure 3 when using the Bayesian approach, or the univariate Cox scores. The univariate Cox scores can lead to very high false discovery rates because the features with non-zero coefficients are usually ranked very low. Furthermore, as shown in Figure 3, the success rates of selecting features with non-zero coefficients are very low even when a large number of features are selected. On the other hand, when more than 12 features are selected using the Bayesian approach, the success rates are usually higher than 0.8 and approach 1 very quickly as more features are selected.

### Application to a real dataset

We applied the proposed two-stage procedure to data on diffuse large B-cell lymphoma (DLBCL) [15]. These data include the expression levels of 7, 399 genes from a total of 240 patients. The genomic information for each patient was obtained at the beginning of the study, and the patients were followed up until death or the end of the project. The missing gene expression values were imputed using the nearest neighbor averaging approach [12,23]. Using the single linkage clustering approach in Cluster 3.0 [24], we identified 5,656 linkage clusters by pruning the hierarchical tree such that the node distances within branches are less than 0.2. There are 4,944 linkage clusters containing only one gene, while the largest has 186 genes. We then consider selecting prognostic molecular features from the 5, 656 candidates, each being the centroid of a linkage cluster.

**Figure 4:**
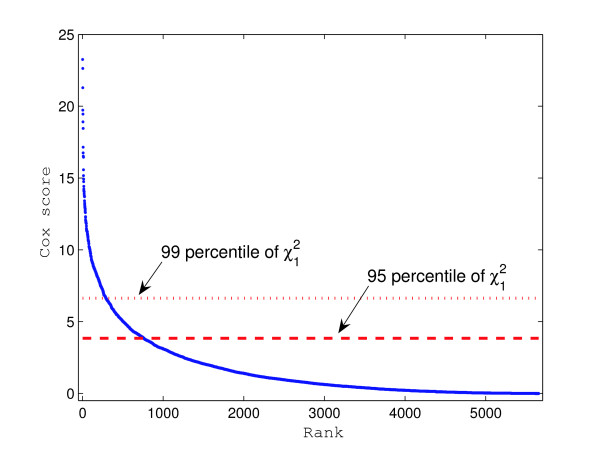
**Cox Score plot for DLBCL data**. This figure shows the descending Cox scores of 5,656 Candidates in the DLBCL data. The dotted and dashed lines indicate the 99 and 95 percentiles of the χ12
 MathType@MTEF@5@5@+=feaafiart1ev1aaatCvAUfKttLearuWrP9MDH5MBPbIqV92AaeXatLxBI9gBaebbnrfifHhDYfgasaacH8akY=wiFfYdH8Gipec8Eeeu0xXdbba9frFj0=OqFfea0dXdd9vqai=hGuQ8kuc9pgc9s8qqaq=dirpe0xb9q8qiLsFr0=vr0=vr0dc8meaabaqaciaacaGaaeqabaqabeGadaaakeaaiiGacqWFhpWydaqhaaWcbaGaeGymaedabaGaeGOmaidaaaaa@3079@ distribution respectively.

The univariate Cox scores of all candidate clusters are calculated and shown in decreasing order in Figure 4. There are 761 candidates with Cox scores above the 95 percentile of the χ12
 MathType@MTEF@5@5@+=feaafiart1ev1aaatCvAUfKttLearuWrP9MDH5MBPbIqV92AaeXatLxBI9gBaebbnrfifHhDYfgasaacH8akY=wiFfYdH8Gipec8Eeeu0xXdbba9frFj0=OqFfea0dXdd9vqai=hGuQ8kuc9pgc9s8qqaq=dirpe0xb9q8qiLsFr0=vr0=vr0dc8meaabaqaciaacaGaaeqabaqabeGadaaakeaaiiGacqWFhpWydaqhaaWcbaGaeGymaedabaGaeGOmaidaaaaa@3079@ distribution, and 290 candidates with Cox scores above the 99 percentile of the χ12
 MathType@MTEF@5@5@+=feaafiart1ev1aaatCvAUfKttLearuWrP9MDH5MBPbIqV92AaeXatLxBI9gBaebbnrfifHhDYfgasaacH8akY=wiFfYdH8Gipec8Eeeu0xXdbba9frFj0=OqFfea0dXdd9vqai=hGuQ8kuc9pgc9s8qqaq=dirpe0xb9q8qiLsFr0=vr0=vr0dc8meaabaqaciaacaGaaeqabaqabeGadaaakeaaiiGacqWFhpWydaqhaaWcbaGaeGymaedabaGaeGOmaidaaaaa@3079@ distribution. We selected the top 100, 200, 300, and 500 candidates with the largest Cox scores and applied our Bayesian method to profile them. The top 25 of the 500 candidates are listed in Table 1.

**Table 1:** Bayesian Profiling of the DLBCL Data. The 16 starred genes are proposed by our exploratory selection. Bracketed are the numbers of features from the same gene, which are included in the same cluster.

GenBank Accession No.	Cox		100		200		300		500	
					
	Score	Rank	p˜ MathType@MTEF@5@5@+=feaafiart1ev1aaatCvAUfKttLearuWrP9MDH5MBPbIqV92AaeXatLxBI9gBaebbnrfifHhDYfgasaacH8akY=wiFfYdH8Gipec8Eeeu0xXdbba9frFj0=OqFfea0dXdd9vqai=hGuQ8kuc9pgc9s8qqaq=dirpe0xb9q8qiLsFr0=vr0=vr0dc8meaabaqaciaacaGaaeqabaqabeGadaaakeaacuWGWbaCgaacaaaa@2E24@_*k*_	Rank	p˜ MathType@MTEF@5@5@+=feaafiart1ev1aaatCvAUfKttLearuWrP9MDH5MBPbIqV92AaeXatLxBI9gBaebbnrfifHhDYfgasaacH8akY=wiFfYdH8Gipec8Eeeu0xXdbba9frFj0=OqFfea0dXdd9vqai=hGuQ8kuc9pgc9s8qqaq=dirpe0xb9q8qiLsFr0=vr0=vr0dc8meaabaqaciaacaGaaeqabaqabeGadaaakeaacuWGWbaCgaacaaaa@2E24@_*k*_	Rank	p˜ MathType@MTEF@5@5@+=feaafiart1ev1aaatCvAUfKttLearuWrP9MDH5MBPbIqV92AaeXatLxBI9gBaebbnrfifHhDYfgasaacH8akY=wiFfYdH8Gipec8Eeeu0xXdbba9frFj0=OqFfea0dXdd9vqai=hGuQ8kuc9pgc9s8qqaq=dirpe0xb9q8qiLsFr0=vr0=vr0dc8meaabaqaciaacaGaaeqabaqabeGadaaakeaacuWGWbaCgaacaaaa@2E24@_*k*_	Rank	p˜ MathType@MTEF@5@5@+=feaafiart1ev1aaatCvAUfKttLearuWrP9MDH5MBPbIqV92AaeXatLxBI9gBaebbnrfifHhDYfgasaacH8akY=wiFfYdH8Gipec8Eeeu0xXdbba9frFj0=OqFfea0dXdd9vqai=hGuQ8kuc9pgc9s8qqaq=dirpe0xb9q8qiLsFr0=vr0=vr0dc8meaabaqaciaacaGaaeqabaqabeGadaaakeaacuWGWbaCgaacaaaa@2E24@_*k*_	Rank
*D42043	18.91	6	0.99	2	0.99	1	0.99	1	0.98	1
*D88532	9.13	122	-	-	0.99	2	0.89	2	0.93	2
*U50196	6.82	272	-	-	-	-	0.12	6	0.32	3
*BC012161	21.28	3	0.81	4	0.55	4	0.61	4	0.31	4
*AF414120	5.77	387	-	-	-	-	-	-	0.27	5
*AF004709	5.27	460	-	-	-	-	-	-	0.20	6
AF127481	17.14	8	1.00	1	0.90	3	0.67	3	0.19	7
*AK025954	5.93	365	-	-	-	-	-	-	0.14	8
*AA504484 (2)	5.23	468	-	-	-	-	-	-	0.11	9
J00220	8.40	167	-	-	0.32	9	0.09	11	0.09	10
*AA837319	9.84	96	0.45	6	0.20	11	0.07	15	0.09	11
AA027985	11.44	50	0.30	9	0.18	13	0.09	10	0.09	12
*D13666	12.85	32	0.34	8	0.34	7	0.10	7	0.08	13
LC_33732	7.52	223	-	-	-	-	0.07	16	0.08	14
*AK000271	7.01	260	-	-	-	-	0.09	12	0.08	15
AF134159	14.11	18	0.43	7	0.20	10	0.08	13	0.07	16
*AA805749	5.72	393	-	-	-	-	-	-	0.07	17
*U46767	11.33	52	0.51	5	0.20	12	0.13	5	0.06	18
*AA804793	9.21	117	-	-	0.43	6	0.09	9	0.06	19
AA829241	12.76	33	0.25	10	0.12	21	0.06	19	0.06	20
*NM_000176 (2)	11.28	56	0.19	12	0.13	18	0.02	61	0.06	21
X00452 (5)	14.42	16	0.92	3	0.46	5	0.10	8	0.06	22
X00457 (3)										
X62744 (3)										
U15085 (4)										
M16276 (4)										
K01171 (5)										
M20430 (4)										
M83664 (2)										
K01144 (6)										
AK000170										
LC_24239										
*X52186	5.44	436	-	-	-	-	-	-	0.05	23
U59302	6.68	287	-	-	-	-	0.07	14	0.05	24
AA825732	7.67	215	-	-	-	-	0.05	21	0.05	25

Employing our Bayesian approach to profile the 500 candidates with the largest Cox scores, the posterior probabilities, i.e., p˜
 MathType@MTEF@5@5@+=feaafiart1ev1aaatCvAUfKttLearuWrP9MDH5MBPbIqV92AaeXatLxBI9gBaebbnrfifHhDYfgasaacH8akY=wiFfYdH8Gipec8Eeeu0xXdbba9frFj0=OqFfea0dXdd9vqai=hGuQ8kuc9pgc9s8qqaq=dirpe0xb9q8qiLsFr0=vr0=vr0dc8meaabaqaciaacaGaaeqabaqabeGadaaakeaacuWGWbaCgaacaaaa@2E24@_*k *_defined in (2), of the top 25 clusters range from 0.0538 to 0.9825. However, the ranks of these 25 clusters vary widely when their univariate Cox scores are used, and only five of those with the top 25 univariate Cox scores appear in this list. Therefore, it may be misleading to profile the clusters for their prognostic ability on the basis of their univariate Cox scores, since many false prognostic features can be highly ranked owing to the complicated correlation structure among features.

When fewer than 500 candidates, for example, 100, 200 or 300, are profiled with the Bayesian approach, most of those that appeared in the top 25 of the 500 profiled candidates are also among the top 25 clusters as long as they are profiled. Indeed, the only exception is the cluster with two features in gene NM_00176, which was ranked at 61 when 300 candidates were profiled by the Bayesian approach. However, the complicated correlation structure between clusters makes it preferable to profile a number of clusters sufficient to avoid missing critical prognostic features. An exploratory selection of prognostic features from the top 25 clusters shown in Table 1 implies that 16 genes may be considered to construct prognostic features for the event time, and some of these features were ignored from the lists of 100, 200, and 300 candidates chosen on the basis of their univariate Cox scores. The cluster with 38 features from 11 genes is not one of the 16 selected, though all those genes except AK000170 belong to the MHC class II signature group defined by Rosenwald et al. [15]. D13666, which was reported by both Sha et al. [20] and Gui and Li [25], belongs to the lymph-node signature group, and BC012161 and AF134159 belong to the proliferation signature group (see Rosenwald et al. [15]). D42043, D88532, BC012161, and LC_33732 were also reported by Sha et al. [20]. It is interesting to observe that, among the 16 selected genes, AF414120 (gene CTLA4) is a member of the immunoglobulin superfamily and encodes a protein that transmits an inhibitory signal to T cells (Ling et al. [26]). AF127481, a lymphoid blast crisis oncogene (LBC), plays an important role in regulating the Rho/Rac GTPase cycle while the Rho/Rac family of small GTPases mediates cytoskeletal reorganization, gene transcription, and cell cycle progression through unique signal transduction pathways (Sterpetti et al. [27]). U46767 (gene CCL13) encodes a cytokine that plays a role in the accumulation of leukocytes during inflammation (Garcia-Zepeda et al. [28]). NM_000176 (gene NR3C1) encodes a receptor for glucocorticoids that can act as both a transcription factor and a regulator of other transcription factors. This protein can also be found in heteromeric cytoplasmic complexes along with heat shock factors and immunophilins (Subramaniam et al. [29]). X52186 (gene ITGB4) encodes the integrin beta 4 subunit, a receptor for the laminins, which tends to associate with the alpha 6 subunit and is likely to play a pivotal role in the biology of invasive carcinoma (Hogervorst et al. [30]).

## Discussion

With high-throughput techniques now available, there has been extensive recent discussion of disease-specific molecular signatures [31,32]. The whole genome and proteome profiles for each of the limited number of patients presents an enormous number of molecular candidates with a complicated correlation structure. Here we group highly-correlated molecular features into linkage clusters in order to profile prognostic signatures. While the molecular features within the same linkage clusters are expected to have similar prognostic association with the event time, physical linkages and metabolic pathways will be able to provide confirmatory information. Thereupon, we strongly suggest that available genome, proteome, and metaboleme information be explored and combined with observed profiles to construct the linkage clusters. By doing this, we can improve the reliability significantly and establish the biological functionality of linkage clusters without overusing the limited number of profiles.

When an optimal subset with a prespecified number of candidates is targeted, classical model selection approaches may be employed to explore all possible subsets and identify the best one. However, "small *n *large *p*" datasets may still obstruct this practice because of the enormous computation and unidentifiable models involved. Indeed, when *p *diverges as *n *→ ∞, many classical approaches may not work even if *p *<*n*. Although univariate Cox scores are frequently utilized to profile candidates and accordingly select the subset, it is risky to identify prognostic signatures by this approach as it is easy to include false signatures but miss the true ones owing to the strong correlations among molecular features. For example, when a molecular feature is positively correlated with both true signatures, it may happen that the false one, instead of the two true ones, is selected. Ein-Dor et al. [33] discussed the discrepancies while using a univariate approach. Built upon the multivariate proportional hazard model (1), the proposed Bayesian approach is able to search all possible subsets of a certain size stochastically via Gibbs sampling. With restrictive priors for "small *n *large *p*" datasets, the posterior probability p˜
 MathType@MTEF@5@5@+=feaafiart1ev1aaatCvAUfKttLearuWrP9MDH5MBPbIqV92AaeXatLxBI9gBaebbnrfifHhDYfgasaacH8akY=wiFfYdH8Gipec8Eeeu0xXdbba9frFj0=OqFfea0dXdd9vqai=hGuQ8kuc9pgc9s8qqaq=dirpe0xb9q8qiLsFr0=vr0=vr0dc8meaabaqaciaacaGaaeqabaqabeGadaaakeaacuWGWbaCgaacaaaa@2E24@_*k *_serves as a relative measure for profiling each candidate's prognostic association with the event time, accounting for other candidates. It is straightforward to extend this Bayesian approach to profile molecular signatures by controlling other clinical factors [3] and considering microenvironments [34].

The three-component prior for the coefficients in model (1) is crucial in constructing the profiling criterion. First, the prior probability of each component can be controlled with a uniform distribution on a subset of [0,1] to guarantee that the model is identifiable such that a Gibbs sampler can feasibly be employed to search the parameter space stochastically. Putting these prior probabilities on a restricted interval allows various numbers of nonzero coefficients in model (1). Second, the three-component prior approach provides flexibility in the possible imbalance between the scales and/or sizes of positive and negative coefficients in the model. Third, the three-component prior automatically results in a three-component posterior distribution for each coefficient, with the posterior probability of each component available for further calculation. In summary, the profiling criterion, posterior probability (p˜
 MathType@MTEF@5@5@+=feaafiart1ev1aaatCvAUfKttLearuWrP9MDH5MBPbIqV92AaeXatLxBI9gBaebbnrfifHhDYfgasaacH8akY=wiFfYdH8Gipec8Eeeu0xXdbba9frFj0=OqFfea0dXdd9vqai=hGuQ8kuc9pgc9s8qqaq=dirpe0xb9q8qiLsFr0=vr0=vr0dc8meaabaqaciaacaGaaeqabaqabeGadaaakeaacuWGWbaCgaacaaaa@2E24@_*k*_), has a natural explanation and can be easily implemented in practice.

## Methods

### Construction of linkage clusters and their centroids

**Figure 5:**
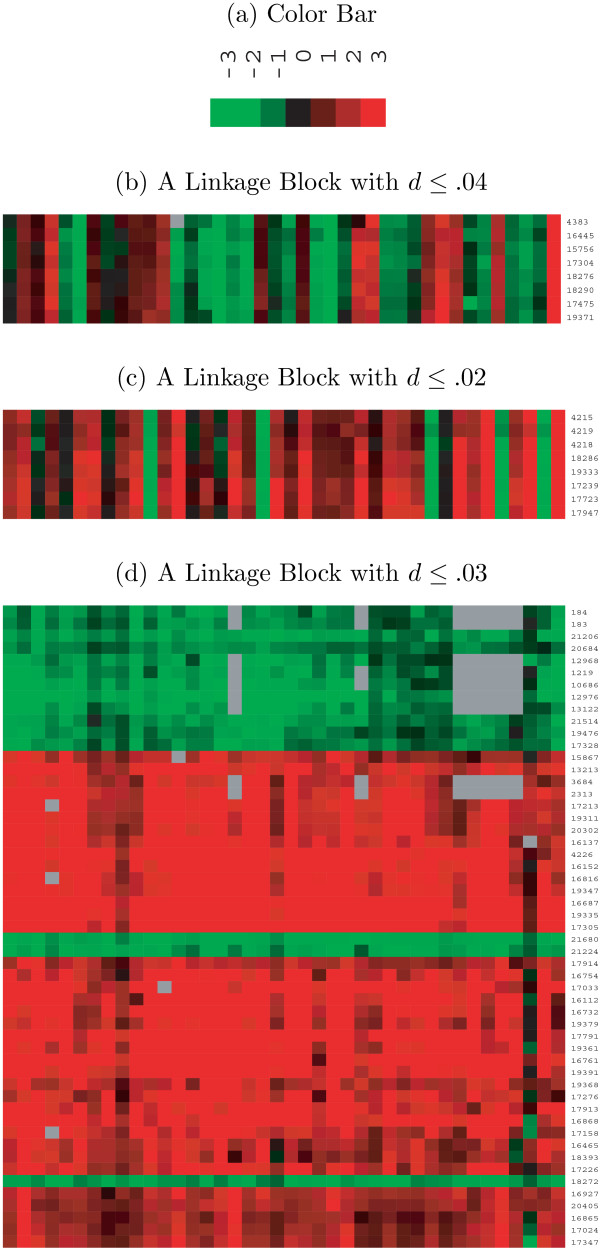
**Illustration of Linkage Blocks**. Part (a) indicates the color bar used in the other parts. Parts (b), (c) and (d) represent three different linkage blocks with correlation coefficients being 0.96, 0.98, and 0.97 respectively, where each row corresponds to a gene and each column corresponds to an individual.

To facilitate pattern recognition and reveal otherwise hidden structures and functions, genes and proteins are usually clustered into groups based on different biological metrics, such as sequence similarity [35] or expression profiles [12,36]. With gene expression data only, many approaches have been applied to cluster genes that exhibit similar expression profiles across samples (see the review by Jörnsten and Yu, [36]). As we are interested in the prognostic relationships of genes to the event time, highly correlated genes are more likely to have the same power to predict the event time and therefore should be grouped into the same cluster. Here we define linkage clusters as groups of genes with large pairwise correlations in absolute value. As shown by Shaffer et al. [37], these linkage clusters of molecular features may reveal functional similarity/dissimilarity. Proteins regulated in the same metabolic pathway may also act similarly or oppositely. Although expressions of genes/proteins are usually observed with measurement errors, these linkage clusters can still be identified in experimental data. As shown in Figure 5, the molecular features can be mutually correlated as highly as correlation coefficient |*ρ*| ≥ 0.96. From a statistical point of view, these closely-related molecular features can cause collinearity or near collinearity in multivariate identification of prognostic signatures and therefore destabilize the identification result if all these closely-related molecular features are included in the prognostic model.

Hierarchical clustering approaches (e.g., the complete linkage clustering or single linkage clustering approach in Cluster 3.0 by de Hoon et al. [24]) can be used to construct these linkage clusters. With the mutual correlation coefficients estimated from the data, we use the absolute values of correlation coefficients as the similarity scores, i.e., with the distance measure *d *= 1 - |*ρ*|. We prune the hierarchical tree with a prespecified value for the distance, e.g., *d *≤ 0.2 (hence absolute values of the correlation coefficients are no less than 0.8). The molecular features within the same branch are assumed to be within the same linkage cluster. The centroid of the linkage cluster is used to represent all the elements within the linkage cluster, and subsequent identification of prognostic signatures proceeds by associating these centroids only with the event time.

The expression levels of the centroids are calculated by standardizing expressions of all genes. More specifically, for each linkage cluster, we first randomly select one gene and reverse the expression signs of all genes within the cluster that are negatively correlated with this gene. Then the expression level of the centroid is calculated by averaging the expression levels of all the genes within the same linkage cluster. Meanwhile, the measurement errors in expression levels are attenuated after averaging the gene expression levels within the same linkage cluster.

### Bayesian framework of proportional hazard model

Suppose that *p *linkage clusters are identified and therefore the expressions of the *p *centroids are calculated for each of the *n *individuals. The observed event time for the *i*-th individual is denoted *y*_*i*_, with *δ*_*i *_indicating whether it was right-censored owing to loss in follow-up (i.e., *δ*_*i *_= 0 if right-censored and *δ*_*i *_= 1 otherwise). Accordingly, the expressions of the centroids are denoted ***z***_*i *_= (*z*_*i*1_, *z*_*i*2_, …, *z*_*ip*_). We use the popular Proportional Hazard Model [4] to associate the molecular features with the event time, i.e., the hazard function is modeled as follows:

*λ*(*t*|***z***_*i*_) = *λ*_0_(*t*)exp(***z***_*i *_***β***),     (1)

where ***β ***includes the *p *coefficients for all the centroids, and *λ*_0_(·) is an unspecified baseline hazard function.

Further, let D
 MathType@MTEF@5@5@+=feaafiart1ev1aaatCvAUfKttLearuWrP9MDH5MBPbIqV92AaeXatLxBI9gBamrtHrhAL1wy0L2yHvtyaeHbnfgDOvwBHrxAJfwnaebbnrfifHhDYfgasaacH8akY=wiFfYdH8Gipec8Eeeu0xXdbba9frFj0=OqFfea0dXdd9vqai=hGuQ8kuc9pgc9s8qqaq=dirpe0xb9q8qiLsFr0=vr0=vr0dc8meaabaqaciaacaGaaeqabaWaaeGaeaaakeaaimaacqWFdepraaa@3827@ = {(*y*_*i*_, *δ*_*i*_, *z*_*i*_) : *i *= 1, 2, …, *n*} be the observed data, and ℛ
 MathType@MTEF@5@5@+=feaafiart1ev1aaatCvAUfKttLearuWrP9MDH5MBPbIqV92AaeXatLxBI9gBamrtHrhAL1wy0L2yHvtyaeHbnfgDOvwBHrxAJfwnaebbnrfifHhDYfgasaacH8akY=wiFfYdH8Gipec8Eeeu0xXdbba9frFj0=OqFfea0dXdd9vqai=hGuQ8kuc9pgc9s8qqaq=dirpe0xb9q8qiLsFr0=vr0=vr0dc8meaabaqaciaacaGaaeqabaWaaeGaeaaakeaaimaacqWFBeIuaaa@377D@(*t*) = {*i *: *y*_*i *_≥ *t*} be the risk set at time *t*. Following Kalbfleisch [22], we construct the Bayesian framework to estimate ***β ***by considering only the partial likelihood function,

PL(β|D)=∏i=1n{exp⁡{ziβ}∑j∈ℛ(yi)exp⁡{zjβ}}δi,
 MathType@MTEF@5@5@+=feaafiart1ev1aaatCvAUfKttLearuWrP9MDH5MBPbIqV92AaeXatLxBI9gBaebbnrfifHhDYfgasaacH8akY=wiFfYdH8Gipec8Eeeu0xXdbba9frFj0=OqFfea0dXdd9vqai=hGuQ8kuc9pgc9s8qqaq=dirpe0xb9q8qiLsFr0=vr0=vr0dc8meaabaqaciaacaGaaeqabaqabeGadaaakeaacqWGqbaucqWGmbatcqGGOaakiiWacqWFYoGycqGG8baFt0uy0HwzTfgDPnwy1egaryqtHrhAL1wy0L2yHvdaiqaacqGFdeprcqGGPaqkcqGH9aqpdaqeWbqaamaacmqabaWaaSaaaeaacyGGLbqzcqGG4baEcqGGWbaCdaGadeqaaGqadiab9Pha6naaBaaaleaacqWGPbqAaeqaaOGae8NSdigacaGL7bGaayzFaaaabaWaaabeaeaacyGGLbqzcqGG4baEcqGGWbaCdaGadeqaaiab9Pha6naaBaaaleaacqWGQbGAaeqaaOGae8NSdigacaGL7bGaayzFaaaaleaacqWGQbGAcqGHiiIZcqGFBeIucqGGOaakcqWG5bqEdaWgaaadbaGaemyAaKgabeaaliabcMcaPaqab0GaeyyeIuoaaaaakiaawUhacaGL9baaaSqaaiabdMgaPjabg2da9iabigdaXaqaaiabd6gaUbqdcqGHpis1aOWaaWbaaSqabeaaiiGacqaF0oazdaWgaaadbaGaemyAaKgabeaaaaGccqGGSaalaaa@6E8D@

which avoids the nuisance baseline hazard function *λ*_0_(*t*).

With a large number of available linkage clusters, the time-to-event of interest may be associated with a relatively small number of linkage clusters. On the other hand, the available "large *p *small *n*" data sets hamper us in detecting linkage clusters with too weak effects on the time-to-event of interest, and we expect to be able to identify those linkage clusters with strong effects. We therefore incorporate this important prior information by considering the following prior distribution for each *β*_*k*_:

*β_k _*~ (1 - *w*_+ _- *w*_-_)*δ*_{0} _+ *w*_+_*N*_+_(0, σ+2
 MathType@MTEF@5@5@+=feaafiart1ev1aaatCvAUfKttLearuWrP9MDH5MBPbIqV92AaeXatLxBI9gBaebbnrfifHhDYfgasaacH8akY=wiFfYdH8Gipec8Eeeu0xXdbba9frFj0=OqFfea0dXdd9vqai=hGuQ8kuc9pgc9s8qqaq=dirpe0xb9q8qiLsFr0=vr0=vr0dc8meaabaqaciaacaGaaeqabaqabeGadaaakeaaiiGacqWFdpWCdaqhaaWcbaGaey4kaScabaGaeGOmaidaaaaa@3077@) + *w*_-_*N*_-_(0, σ−2
 MathType@MTEF@5@5@+=feaafiart1ev1aaatCvAUfKttLearuWrP9MDH5MBPbIqV92AaeXatLxBI9gBaebbnrfifHhDYfgasaacH8akY=wiFfYdH8Gipec8Eeeu0xXdbba9frFj0=OqFfea0dXdd9vqai=hGuQ8kuc9pgc9s8qqaq=dirpe0xb9q8qiLsFr0=vr0=vr0dc8meaabaqaciaacaGaaeqabaqabeGadaaakeaaiiGacqWFdpWCdaqhaaWcbaGaeyOeI0cabaGaeGOmaidaaaaa@3082@),

where *N*_+_(*μ*, *σ*^2^) and *N*_-_(*μ*, *σ*^2^) are the truncated Gaussian distributions with only positive and negative parts, respectively. As shown by Zhang et al. [38] and Zhang et al. [39], this three-component prior has some theoretical properties and allows a possible imbalance between scales and/or sizes of positive and negative coefficients in model (1).

*A priori*, the hyperparameters σ+2
 MathType@MTEF@5@5@+=feaafiart1ev1aaatCvAUfKttLearuWrP9MDH5MBPbIqV92AaeXatLxBI9gBaebbnrfifHhDYfgasaacH8akY=wiFfYdH8Gipec8Eeeu0xXdbba9frFj0=OqFfea0dXdd9vqai=hGuQ8kuc9pgc9s8qqaq=dirpe0xb9q8qiLsFr0=vr0=vr0dc8meaabaqaciaacaGaaeqabaqabeGadaaakeaaiiGacqWFdpWCdaqhaaWcbaGaey4kaScabaGaeGOmaidaaaaa@3077@ and σ−2
 MathType@MTEF@5@5@+=feaafiart1ev1aaatCvAUfKttLearuWrP9MDH5MBPbIqV92AaeXatLxBI9gBaebbnrfifHhDYfgasaacH8akY=wiFfYdH8Gipec8Eeeu0xXdbba9frFj0=OqFfea0dXdd9vqai=hGuQ8kuc9pgc9s8qqaq=dirpe0xb9q8qiLsFr0=vr0=vr0dc8meaabaqaciaacaGaaeqabaqabeGadaaakeaaiiGacqWFdpWCdaqhaaWcbaGaeyOeI0cabaGaeGOmaidaaaaa@3082@ are assumed to follow inverse gamma distributions as *IG*(1, *φ*_+_) and *IG*(1, *φ*_-_), respectively. Here, sufficiently large *φ*_+ _and *φ*_- _are recommended to approximate the noninformative priors 1/σ+2
 MathType@MTEF@5@5@+=feaafiart1ev1aaatCvAUfKttLearuWrP9MDH5MBPbIqV92AaeXatLxBI9gBaebbnrfifHhDYfgasaacH8akY=wiFfYdH8Gipec8Eeeu0xXdbba9frFj0=OqFfea0dXdd9vqai=hGuQ8kuc9pgc9s8qqaq=dirpe0xb9q8qiLsFr0=vr0=vr0dc8meaabaqaciaacaGaaeqabaqabeGadaaakeaaiiGacqWFdpWCdaqhaaWcbaGaey4kaScabaGaeGOmaidaaaaa@3077@ and 1/σ−2
 MathType@MTEF@5@5@+=feaafiart1ev1aaatCvAUfKttLearuWrP9MDH5MBPbIqV92AaeXatLxBI9gBaebbnrfifHhDYfgasaacH8akY=wiFfYdH8Gipec8Eeeu0xXdbba9frFj0=OqFfea0dXdd9vqai=hGuQ8kuc9pgc9s8qqaq=dirpe0xb9q8qiLsFr0=vr0=vr0dc8meaabaqaciaacaGaaeqabaqabeGadaaakeaaiiGacqWFdpWCdaqhaaWcbaGaeyOeI0cabaGaeGOmaidaaaaa@3082@, respectively. For the prior distribution of (*w*_+_,*w*_-_), a noninformative prior (such as *Dirichlet*(1, 1, 1)) is not applicable since the model is not identifiable with *p *» *n*. As shown in Zhang et al. [40], the number of reliably identified significant predictors is limited by the sample size *n*. Following Zhang et al. [39] and Zhang et al. [41], we let

*w*_+ _+ *w*_- _~ *Unif*(0, 2n
 MathType@MTEF@5@5@+=feaafiart1ev1aaatCvAUfKttLearuWrP9MDH5MBPbIqV92AaeXatLxBI9gBaebbnrfifHhDYfgasaacH8akY=wiFfYdH8Gipec8Eeeu0xXdbba9frFj0=OqFfea0dXdd9vqai=hGuQ8kuc9pgc9s8qqaq=dirpe0xb9q8qiLsFr0=vr0=vr0dc8meaabaqaciaacaGaaeqabaqabeGadaaakeaadaGcaaqaaiabd6gaUbWcbeaaaaa@2E2C@/*p*),

which guarantees the model to be identifiable. On the other hand, as the number of candidate predictors is large, the upper bound, 2n
 MathType@MTEF@5@5@+=feaafiart1ev1aaatCvAUfKttLearuWrP9MDH5MBPbIqV92AaeXatLxBI9gBaebbnrfifHhDYfgasaacH8akY=wiFfYdH8Gipec8Eeeu0xXdbba9frFj0=OqFfea0dXdd9vqai=hGuQ8kuc9pgc9s8qqaq=dirpe0xb9q8qiLsFr0=vr0=vr0dc8meaabaqaciaacaGaaeqabaqabeGadaaakeaadaGcaaqaaiabd6gaUbWcbeaaaaa@2E2C@/*p*, on (*w*_+ _+ *w*_-_) can be so restrictive that the resultant posterior probability for a true predictor to be significant can be very small. However, these posterior probabilities, as relative measures of significance, play an important role in profiling all features for their prognostic relations to the event time.

### The Gibbs sampler

In view of the large number of parameters to be estimated, we consider a Gibbs sampler to obtain the posterior distributions of the parameters and make inferences. The Gibbs sampler can be developed by iteratively sampling each parameter from its full conditional distribution.

For simplicity, let ***β***_-*k *_include all components of ***β ***except *β*_*k*_, and write *g*_*k*_(*β*_*k*_|*β*_-*k*_, D
 MathType@MTEF@5@5@+=feaafiart1ev1aaatCvAUfKttLearuWrP9MDH5MBPbIqV92AaeXatLxBI9gBamrtHrhAL1wy0L2yHvtyaeHbnfgDOvwBHrxAJfwnaebbnrfifHhDYfgasaacH8akY=wiFfYdH8Gipec8Eeeu0xXdbba9frFj0=OqFfea0dXdd9vqai=hGuQ8kuc9pgc9s8qqaq=dirpe0xb9q8qiLsFr0=vr0=vr0dc8meaabaqaciaacaGaaeqabaWaaeGaeaaakeaaimaacqWFdepraaa@3827@) = *PL*(*β*|D
 MathType@MTEF@5@5@+=feaafiart1ev1aaatCvAUfKttLearuWrP9MDH5MBPbIqV92AaeXatLxBI9gBamrtHrhAL1wy0L2yHvtyaeHbnfgDOvwBHrxAJfwnaebbnrfifHhDYfgasaacH8akY=wiFfYdH8Gipec8Eeeu0xXdbba9frFj0=OqFfea0dXdd9vqai=hGuQ8kuc9pgc9s8qqaq=dirpe0xb9q8qiLsFr0=vr0=vr0dc8meaabaqaciaacaGaaeqabaWaaeGaeaaakeaaimaacqWFdepraaa@3827@) when *β*_*k *_is of particular interest. Then the full conditional distribution of *β*_*k *_is a mixture of a point mass at zero and two continuous distributions, i.e.,

*β*_*k*_|*w*_+_, *w*_-_,σ+2
 MathType@MTEF@5@5@+=feaafiart1ev1aaatCvAUfKttLearuWrP9MDH5MBPbIqV92AaeXatLxBI9gBaebbnrfifHhDYfgasaacH8akY=wiFfYdH8Gipec8Eeeu0xXdbba9frFj0=OqFfea0dXdd9vqai=hGuQ8kuc9pgc9s8qqaq=dirpe0xb9q8qiLsFr0=vr0=vr0dc8meaabaqaciaacaGaaeqabaqabeGadaaakeaaiiGacqWFdpWCdaqhaaWcbaGaey4kaScabaGaeGOmaidaaaaa@3077@,σ−2
 MathType@MTEF@5@5@+=feaafiart1ev1aaatCvAUfKttLearuWrP9MDH5MBPbIqV92AaeXatLxBI9gBaebbnrfifHhDYfgasaacH8akY=wiFfYdH8Gipec8Eeeu0xXdbba9frFj0=OqFfea0dXdd9vqai=hGuQ8kuc9pgc9s8qqaq=dirpe0xb9q8qiLsFr0=vr0=vr0dc8meaabaqaciaacaGaaeqabaqabeGadaaakeaaiiGacqWFdpWCdaqhaaWcbaGaeyOeI0cabaGaeGOmaidaaaaa@3082@,D
 MathType@MTEF@5@5@+=feaafiart1ev1aaatCvAUfKttLearuWrP9MDH5MBPbIqV92AaeXatLxBI9gBamrtHrhAL1wy0L2yHvtyaeHbnfgDOvwBHrxAJfwnaebbnrfifHhDYfgasaacH8akY=wiFfYdH8Gipec8Eeeu0xXdbba9frFj0=OqFfea0dXdd9vqai=hGuQ8kuc9pgc9s8qqaq=dirpe0xb9q8qiLsFr0=vr0=vr0dc8meaabaqaciaacaGaaeqabaWaaeGaeaaakeaaimaacqWFdepraaa@3827@

~ (1 - w˜
 MathType@MTEF@5@5@+=feaafiart1ev1aaatCvAUfKttLearuWrP9MDH5MBPbIqV92AaeXatLxBI9gBaebbnrfifHhDYfgasaacH8akY=wiFfYdH8Gipec8Eeeu0xXdbba9frFj0=OqFfea0dXdd9vqai=hGuQ8kuc9pgc9s8qqaq=dirpe0xb9q8qiLsFr0=vr0=vr0dc8meaabaqaciaacaGaaeqabaqabeGadaaakeaacuWG3bWDgaacaaaa@2E32@_*k*+ _- w˜
 MathType@MTEF@5@5@+=feaafiart1ev1aaatCvAUfKttLearuWrP9MDH5MBPbIqV92AaeXatLxBI9gBaebbnrfifHhDYfgasaacH8akY=wiFfYdH8Gipec8Eeeu0xXdbba9frFj0=OqFfea0dXdd9vqai=hGuQ8kuc9pgc9s8qqaq=dirpe0xb9q8qiLsFr0=vr0=vr0dc8meaabaqaciaacaGaaeqabaqabeGadaaakeaacuWG3bWDgaacaaaa@2E32@_*k*-_)*δ*_{0} _+ w˜
 MathType@MTEF@5@5@+=feaafiart1ev1aaatCvAUfKttLearuWrP9MDH5MBPbIqV92AaeXatLxBI9gBaebbnrfifHhDYfgasaacH8akY=wiFfYdH8Gipec8Eeeu0xXdbba9frFj0=OqFfea0dXdd9vqai=hGuQ8kuc9pgc9s8qqaq=dirpe0xb9q8qiLsFr0=vr0=vr0dc8meaabaqaciaacaGaaeqabaqabeGadaaakeaacuWG3bWDgaacaaaa@2E32@_*k*+ _F˜
 MathType@MTEF@5@5@+=feaafiart1ev1aaatCvAUfKttLearuWrP9MDH5MBPbIqV92AaeXatLxBI9gBaebbnrfifHhDYfgasaacH8akY=wiFfYdH8Gipec8Eeeu0xXdbba9frFj0=OqFfea0dXdd9vqai=hGuQ8kuc9pgc9s8qqaq=dirpe0xb9q8qiLsFr0=vr0=vr0dc8meaabaqaciaacaGaaeqabaqabeGadaaakeaacuWGgbGrgaacaaaa@2DD0@_*k*+ _+ w˜
 MathType@MTEF@5@5@+=feaafiart1ev1aaatCvAUfKttLearuWrP9MDH5MBPbIqV92AaeXatLxBI9gBaebbnrfifHhDYfgasaacH8akY=wiFfYdH8Gipec8Eeeu0xXdbba9frFj0=OqFfea0dXdd9vqai=hGuQ8kuc9pgc9s8qqaq=dirpe0xb9q8qiLsFr0=vr0=vr0dc8meaabaqaciaacaGaaeqabaqabeGadaaakeaacuWG3bWDgaacaaaa@2E32@_*k*- _F˜
 MathType@MTEF@5@5@+=feaafiart1ev1aaatCvAUfKttLearuWrP9MDH5MBPbIqV92AaeXatLxBI9gBaebbnrfifHhDYfgasaacH8akY=wiFfYdH8Gipec8Eeeu0xXdbba9frFj0=OqFfea0dXdd9vqai=hGuQ8kuc9pgc9s8qqaq=dirpe0xb9q8qiLsFr0=vr0=vr0dc8meaabaqaciaacaGaaeqabaqabeGadaaakeaacuWGgbGrgaacaaaa@2DD0@_*k*-_,

where F˜
 MathType@MTEF@5@5@+=feaafiart1ev1aaatCvAUfKttLearuWrP9MDH5MBPbIqV92AaeXatLxBI9gBaebbnrfifHhDYfgasaacH8akY=wiFfYdH8Gipec8Eeeu0xXdbba9frFj0=OqFfea0dXdd9vqai=hGuQ8kuc9pgc9s8qqaq=dirpe0xb9q8qiLsFr0=vr0=vr0dc8meaabaqaciaacaGaaeqabaqabeGadaaakeaacuWGgbGrgaacaaaa@2DD0@_*k*+ _and F˜
 MathType@MTEF@5@5@+=feaafiart1ev1aaatCvAUfKttLearuWrP9MDH5MBPbIqV92AaeXatLxBI9gBaebbnrfifHhDYfgasaacH8akY=wiFfYdH8Gipec8Eeeu0xXdbba9frFj0=OqFfea0dXdd9vqai=hGuQ8kuc9pgc9s8qqaq=dirpe0xb9q8qiLsFr0=vr0=vr0dc8meaabaqaciaacaGaaeqabaqabeGadaaakeaacuWGgbGrgaacaaaa@2DD0@_*k*- _zare the distributions corresponding to the following probability density functions,

f˜k+(x)=gk(x|β−k,D)wk+2πσ+2exp⁡{−x22σ+2}I[x>0],
 MathType@MTEF@5@5@+=feaafiart1ev1aaatCvAUfKttLearuWrP9MDH5MBPbIqV92AaeXatLxBI9gBaebbnrfifHhDYfgasaacH8akY=wiFfYdH8Gipec8Eeeu0xXdbba9frFj0=OqFfea0dXdd9vqai=hGuQ8kuc9pgc9s8qqaq=dirpe0xb9q8qiLsFr0=vr0=vr0dc8meaabaqaciaacaGaaeqabaqabeGadaaakeaacuWGMbGzgaacamaaBaaaleaacqWGRbWAcqGHRaWkaeqaaOGaeiikaGIaemiEaGNaeiykaKIaeyypa0ZaaSaaaeaacqWGNbWzdaWgaaWcbaGaem4AaSgabeaakiabcIcaOiabdIha4jabcYha8HGadiab=j7aInaaBaaaleaacqGHsislcqWGRbWAaeqaaOGaeiilaWYenfgDOvwBHrxAJfwnHbqeg0uy0HwzTfgDPnwy1aaceaGae43aXtKaeiykaKcabaGaem4DaC3aaSbaaSqaaiabdUgaRjabgUcaRaqabaGcdaGcaaqaaiabikdaYGGaciab9b8aWjab9n8aZnaaDaaaleaacqGHRaWkaeaacqaIYaGmaaaabeaaaaGccyGGLbqzcqGG4baEcqGGWbaCdaGadeqaaiabgkHiTmaalaaabaGaemiEaG3aaWbaaSqabeaacqaIYaGmaaaakeaacqaIYaGmcqqFdpWCdaqhaaWcbaGaey4kaScabaGaeGOmaidaaaaaaOGaay5Eaiaaw2haaiabdMeajjabcUfaBjabdIha4jabg6da+iabicdaWiabc2faDjabcYcaSaaa@6E04@

f˜k−(x)=gk(x|β−k,D)wk−2πσ−2exp⁡{−x22σ−2}I[x<0],
 MathType@MTEF@5@5@+=feaafiart1ev1aaatCvAUfKttLearuWrP9MDH5MBPbIqV92AaeXatLxBI9gBaebbnrfifHhDYfgasaacH8akY=wiFfYdH8Gipec8Eeeu0xXdbba9frFj0=OqFfea0dXdd9vqai=hGuQ8kuc9pgc9s8qqaq=dirpe0xb9q8qiLsFr0=vr0=vr0dc8meaabaqaciaacaGaaeqabaqabeGadaaakeaacuWGMbGzgaacamaaBaaaleaacqWGRbWAcqGHsislaeqaaOGaeiikaGIaemiEaGNaeiykaKIaeyypa0ZaaSaaaeaacqWGNbWzdaWgaaWcbaGaem4AaSgabeaakiabcIcaOiabdIha4jabcYha8HGadiab=j7aInaaBaaaleaacqGHsislcqWGRbWAaeqaaOGaeiilaWYenfgDOvwBHrxAJfwnHbqeg0uy0HwzTfgDPnwy1aaceaGae43aXtKaeiykaKcabaGaem4DaC3aaSbaaSqaaiabdUgaRjabgkHiTaqabaGcdaGcaaqaaiabikdaYGGaciab9b8aWjab9n8aZnaaDaaaleaacqGHsislaeaacqaIYaGmaaaabeaaaaGccyGGLbqzcqGG4baEcqGGWbaCdaGadeqaaiabgkHiTmaalaaabaGaemiEaG3aaWbaaSqabeaacqaIYaGmaaaakeaacqaIYaGmcqqFdpWCdaqhaaWcbaGaeyOeI0cabaGaeGOmaidaaaaaaOGaay5Eaiaaw2haaiabdMeajjabcUfaBjabdIha4jabgYda8iabicdaWiabc2faDjabcYcaSaaa@6E2C@

and the probabilities for *β*_*k *_to be positive and negative are, respectively,

w˜k+=2w+wk+(1−w+−w−)gk(0|β−k,D)+2w+wk++2w−wk−,
 MathType@MTEF@5@5@+=feaafiart1ev1aaatCvAUfKttLearuWrP9MDH5MBPbIqV92AaeXatLxBI9gBaebbnrfifHhDYfgasaacH8akY=wiFfYdH8Gipec8Eeeu0xXdbba9frFj0=OqFfea0dXdd9vqai=hGuQ8kuc9pgc9s8qqaq=dirpe0xb9q8qiLsFr0=vr0=vr0dc8meaabaqaciaacaGaaeqabaqabeGadaaakeaacuWG3bWDgaacamaaBaaaleaacqWGRbWAcqGHRaWkaeqaaOGaeyypa0ZaaSaaaeaacqaIYaGmcqWG3bWDdaWgaaWcbaGaey4kaScabeaakiabdEha3naaBaaaleaacqWGRbWAcqGHRaWkaeqaaaGcbaGaeiikaGIaeGymaeJaeyOeI0Iaem4DaC3aaSbaaSqaaiabgUcaRaqabaGccqGHsislcqWG3bWDdaWgaaWcbaGaeyOeI0cabeaakiabcMcaPiabdEgaNnaaBaaaleaacqWGRbWAaeqaaOGaeiikaGIaeGimaaJaeiiFaWhccmGae8NSdi2aaSbaaSqaaiabgkHiTiabdUgaRbqabaGccqGGSaalt0uy0HwzTfgDPnwy1egaryqtHrhAL1wy0L2yHvdaiqaacqGFdeprcqGGPaqkcqGHRaWkcqaIYaGmcqWG3bWDdaWgaaWcbaGaey4kaScabeaakiabdEha3naaBaaaleaacqWGRbWAcqGHRaWkaeqaaOGaey4kaSIaeGOmaiJaem4DaC3aaSbaaSqaaiabgkHiTaqabaGccqWG3bWDdaWgaaWcbaGaem4AaSMaeyOeI0cabeaaaaGccqGGSaalaaa@6BFB@

w˜k−=2w−wk−(1−w+−w−)gk(0|β−k,D)+2w+wk++2w−wk−.
 MathType@MTEF@5@5@+=feaafiart1ev1aaatCvAUfKttLearuWrP9MDH5MBPbIqV92AaeXatLxBI9gBaebbnrfifHhDYfgasaacH8akY=wiFfYdH8Gipec8Eeeu0xXdbba9frFj0=OqFfea0dXdd9vqai=hGuQ8kuc9pgc9s8qqaq=dirpe0xb9q8qiLsFr0=vr0=vr0dc8meaabaqaciaacaGaaeqabaqabeGadaaakeaacuWG3bWDgaacamaaBaaaleaacqWGRbWAcqGHsislaeqaaOGaeyypa0ZaaSaaaeaacqaIYaGmcqWG3bWDdaWgaaWcbaGaeyOeI0cabeaakiabdEha3naaBaaaleaacqWGRbWAcqGHsislaeqaaaGcbaGaeiikaGIaeGymaeJaeyOeI0Iaem4DaC3aaSbaaSqaaiabgUcaRaqabaGccqGHsislcqWG3bWDdaWgaaWcbaGaeyOeI0cabeaakiabcMcaPiabdEgaNnaaBaaaleaacqWGRbWAaeqaaOGaeiikaGIaeGimaaJaeiiFaWhccmGae8NSdi2aaSbaaSqaaiabgkHiTiabdUgaRbqabaGccqGGSaalt0uy0HwzTfgDPnwy1egaryqtHrhAL1wy0L2yHvdaiqaacqGFdeprcqGGPaqkcqGHRaWkcqaIYaGmcqWG3bWDdaWgaaWcbaGaey4kaScabeaakiabdEha3naaBaaaleaacqWGRbWAcqGHRaWkaeqaaOGaey4kaSIaeGOmaiJaem4DaC3aaSbaaSqaaiabgkHiTaqabaGccqWG3bWDdaWgaaWcbaGaem4AaSMaeyOeI0cabeaaaaGccqGGUaGlaaa@6C20@

Here, *w*_*k*+ _and *w*_*k*- _are normalization coefficients, which can be calculated as

wk+=∫0∞gk(x|β−k,D)2πσ+2exp⁡{−x22σ+2}dx,
 MathType@MTEF@5@5@+=feaafiart1ev1aaatCvAUfKttLearuWrP9MDH5MBPbIqV92AaeXatLxBI9gBaebbnrfifHhDYfgasaacH8akY=wiFfYdH8Gipec8Eeeu0xXdbba9frFj0=OqFfea0dXdd9vqai=hGuQ8kuc9pgc9s8qqaq=dirpe0xb9q8qiLsFr0=vr0=vr0dc8meaabaqaciaacaGaaeqabaqabeGadaaakeaacqWG3bWDdaWgaaWcbaGaem4AaSMaey4kaScabeaakiabg2da9maapedabaWaaSaaaeaacqWGNbWzdaWgaaWcbaGaem4AaSgabeaakiabcIcaOiabdIha4jabcYha8HGadiab=j7aInaaBaaaleaacqGHsislcqWGRbWAaeqaaOGaeiilaWYenfgDOvwBHrxAJfwnHbqeg0uy0HwzTfgDPnwy1aaceaGae43aXtKaeiykaKcabaWaaOaaaeaacqaIYaGmiiGacqqFapaCcqqFdpWCdaqhaaWcbaGaey4kaScabaGaeGOmaidaaaqabaaaaaqaaiabicdaWaqaaiabg6HiLcqdcqGHRiI8aOGagiyzauMaeiiEaGNaeiiCaa3aaiWabeaacqGHsisldaWcaaqaaiabdIha4naaCaaaleqabaGaeGOmaidaaaGcbaGaeGOmaiJae03Wdm3aa0baaSqaaiabgUcaRaqaaiabikdaYaaaaaaakiaawUhacaGL9baacqWGKbazcqWG4baEcqGGSaalaaa@673B@

wk−=∫−∞0gk(x|β−k,D)2πσ−2exp⁡{−x22σ−2}dx.
 MathType@MTEF@5@5@+=feaafiart1ev1aaatCvAUfKttLearuWrP9MDH5MBPbIqV92AaeXatLxBI9gBaebbnrfifHhDYfgasaacH8akY=wiFfYdH8Gipec8Eeeu0xXdbba9frFj0=OqFfea0dXdd9vqai=hGuQ8kuc9pgc9s8qqaq=dirpe0xb9q8qiLsFr0=vr0=vr0dc8meaabaqaciaacaGaaeqabaqabeGadaaakeaacqWG3bWDdaWgaaWcbaGaem4AaSMaeyOeI0cabeaakiabg2da9maapedabaWaaSaaaeaacqWGNbWzdaWgaaWcbaGaem4AaSgabeaakiabcIcaOiabdIha4jabcYha8HGadiab=j7aInaaBaaaleaacqGHsislcqWGRbWAaeqaaOGaeiilaWYenfgDOvwBHrxAJfwnHbqeg0uy0HwzTfgDPnwy1aaceaGae43aXtKaeiykaKcabaWaaOaaaeaacqaIYaGmiiGacqqFapaCcqqFdpWCdaqhaaWcbaGaeyOeI0cabaGaeGOmaidaaaqabaaaaaqaaiabgkHiTiabg6HiLcqaaiabicdaWaqdcqGHRiI8aOGagiyzauMaeiiEaGNaeiiCaa3aaiWabeaacqGHsisldaWcaaqaaiabdIha4naaCaaaleqabaGaeGOmaidaaaGcbaGaeGOmaiJae03Wdm3aa0baaSqaaiabgkHiTaqaaiabikdaYaaaaaaakiaawUhacaGL9baacqWGKbazcqWG4baEcqGGUaGlaaa@684D@

The full conditional distribution of *w*_+ _and *w*_- _is

(*w*_+_, *w*_-_, 1 - *w*_+ _- *w*_-_)|***β***

~ *Dirichlet*(p˜
 MathType@MTEF@5@5@+=feaafiart1ev1aaatCvAUfKttLearuWrP9MDH5MBPbIqV92AaeXatLxBI9gBaebbnrfifHhDYfgasaacH8akY=wiFfYdH8Gipec8Eeeu0xXdbba9frFj0=OqFfea0dXdd9vqai=hGuQ8kuc9pgc9s8qqaq=dirpe0xb9q8qiLsFr0=vr0=vr0dc8meaabaqaciaacaGaaeqabaqabeGadaaakeaacuWGWbaCgaacaaaa@2E24@_+_, p˜
 MathType@MTEF@5@5@+=feaafiart1ev1aaatCvAUfKttLearuWrP9MDH5MBPbIqV92AaeXatLxBI9gBaebbnrfifHhDYfgasaacH8akY=wiFfYdH8Gipec8Eeeu0xXdbba9frFj0=OqFfea0dXdd9vqai=hGuQ8kuc9pgc9s8qqaq=dirpe0xb9q8qiLsFr0=vr0=vr0dc8meaabaqaciaacaGaaeqabaqabeGadaaakeaacuWGWbaCgaacaaaa@2E24@_-_, *p *- p˜
 MathType@MTEF@5@5@+=feaafiart1ev1aaatCvAUfKttLearuWrP9MDH5MBPbIqV92AaeXatLxBI9gBaebbnrfifHhDYfgasaacH8akY=wiFfYdH8Gipec8Eeeu0xXdbba9frFj0=OqFfea0dXdd9vqai=hGuQ8kuc9pgc9s8qqaq=dirpe0xb9q8qiLsFr0=vr0=vr0dc8meaabaqaciaacaGaaeqabaqabeGadaaakeaacuWGWbaCgaacaaaa@2E24@_+ _- p˜
 MathType@MTEF@5@5@+=feaafiart1ev1aaatCvAUfKttLearuWrP9MDH5MBPbIqV92AaeXatLxBI9gBaebbnrfifHhDYfgasaacH8akY=wiFfYdH8Gipec8Eeeu0xXdbba9frFj0=OqFfea0dXdd9vqai=hGuQ8kuc9pgc9s8qqaq=dirpe0xb9q8qiLsFr0=vr0=vr0dc8meaabaqaciaacaGaaeqabaqabeGadaaakeaacuWGWbaCgaacaaaa@2E24@_-_),    *w*_+ _+ *w*_- _≤ 2np
 MathType@MTEF@5@5@+=feaafiart1ev1aaatCvAUfKttLearuWrP9MDH5MBPbIqV92AaeXatLxBI9gBaebbnrfifHhDYfgasaacH8akY=wiFfYdH8Gipec8Eeeu0xXdbba9frFj0=OqFfea0dXdd9vqai=hGuQ8kuc9pgc9s8qqaq=dirpe0xb9q8qiLsFr0=vr0=vr0dc8meaabaqaciaacaGaaeqabaqabeGadaaakeaadaWcaaqaaiabikdaYmaakaaabaGaemOBa4galeqaaaGcbaGaemiCaahaaaaa@30A1@,

where p˜
 MathType@MTEF@5@5@+=feaafiart1ev1aaatCvAUfKttLearuWrP9MDH5MBPbIqV92AaeXatLxBI9gBaebbnrfifHhDYfgasaacH8akY=wiFfYdH8Gipec8Eeeu0xXdbba9frFj0=OqFfea0dXdd9vqai=hGuQ8kuc9pgc9s8qqaq=dirpe0xb9q8qiLsFr0=vr0=vr0dc8meaabaqaciaacaGaaeqabaqabeGadaaakeaacuWGWbaCgaacaaaa@2E24@_+ _= #{*k *: *β_k _*> 0} and p˜
 MathType@MTEF@5@5@+=feaafiart1ev1aaatCvAUfKttLearuWrP9MDH5MBPbIqV92AaeXatLxBI9gBaebbnrfifHhDYfgasaacH8akY=wiFfYdH8Gipec8Eeeu0xXdbba9frFj0=OqFfea0dXdd9vqai=hGuQ8kuc9pgc9s8qqaq=dirpe0xb9q8qiLsFr0=vr0=vr0dc8meaabaqaciaacaGaaeqabaqabeGadaaakeaacuWGWbaCgaacaaaa@2E24@_- _= #{*k *: *β_k _*< 0}. Finally, the full conditional distribution of σ+2
 MathType@MTEF@5@5@+=feaafiart1ev1aaatCvAUfKttLearuWrP9MDH5MBPbIqV92AaeXatLxBI9gBaebbnrfifHhDYfgasaacH8akY=wiFfYdH8Gipec8Eeeu0xXdbba9frFj0=OqFfea0dXdd9vqai=hGuQ8kuc9pgc9s8qqaq=dirpe0xb9q8qiLsFr0=vr0=vr0dc8meaabaqaciaacaGaaeqabaqabeGadaaakeaaiiGacqWFdpWCdaqhaaWcbaGaey4kaScabaGaeGOmaidaaaaa@3077@ and σ−2
 MathType@MTEF@5@5@+=feaafiart1ev1aaatCvAUfKttLearuWrP9MDH5MBPbIqV92AaeXatLxBI9gBaebbnrfifHhDYfgasaacH8akY=wiFfYdH8Gipec8Eeeu0xXdbba9frFj0=OqFfea0dXdd9vqai=hGuQ8kuc9pgc9s8qqaq=dirpe0xb9q8qiLsFr0=vr0=vr0dc8meaabaqaciaacaGaaeqabaqabeGadaaakeaaiiGacqWFdpWCdaqhaaWcbaGaeyOeI0cabaGaeGOmaidaaaaa@3082@ are

σ+−2|β~Gamma(p˜+2,(1φ++12∑k=1pβk2I[βk>0])−1),
 MathType@MTEF@5@5@+=feaafiart1ev1aaatCvAUfKttLearuWrP9MDH5MBPbIqV92AaeXatLxBI9gBaebbnrfifHhDYfgasaacH8akY=wiFfYdH8Gipec8Eeeu0xXdbba9frFj0=OqFfea0dXdd9vqai=hGuQ8kuc9pgc9s8qqaq=dirpe0xb9q8qiLsFr0=vr0=vr0dc8meaabaqaciaacaGaaeqabaqabeGadaaakeaaiiGacqWFdpWCdaqhaaWcbaGaey4kaScabaGaeyOeI0IaeGOmaidaaOGaeiiFaWhccmGae4NSdiMaeiOFa4Naem4raCKaemyyaeMaemyBa0MaemyBa0Maemyyae2aaeWaaeaadaWcaaqaaiqbdchaWzaaiaWaaSbaaSqaaiabgUcaRaqabaaakeaacqaIYaGmaaGaeiilaWYaaeWaaeaadaWcaaqaaiabigdaXaqaaiab=z8aMnaaBaaaleaacqGHRaWkaeqaaaaakiabgUcaRmaalaaabaGaeGymaedabaGaeGOmaidaamaaqahabaGae8NSdi2aa0baaSqaaiabdUgaRbqaaiabikdaYaaaaeaacqWGRbWAcqGH9aqpcqaIXaqmaeaacqWGWbaCa0GaeyyeIuoakiabdMeajjabcUfaBjab=j7aInaaBaaaleaacqWGRbWAaeqaaOGaeyOpa4JaeGimaaJaeiyxa0facaGLOaGaayzkaaWaaWbaaSqabeaacqGHsislcqaIXaqmaaaakiaawIcacaGLPaaacqGGSaalaaa@6180@

σ−−2|β~Gamma(p˜−2,(1φ−+12∑k=1pβk2I[βk<0])−1).
 MathType@MTEF@5@5@+=feaafiart1ev1aaatCvAUfKttLearuWrP9MDH5MBPbIqV92AaeXatLxBI9gBaebbnrfifHhDYfgasaacH8akY=wiFfYdH8Gipec8Eeeu0xXdbba9frFj0=OqFfea0dXdd9vqai=hGuQ8kuc9pgc9s8qqaq=dirpe0xb9q8qiLsFr0=vr0=vr0dc8meaabaqaciaacaGaaeqabaqabeGadaaakeaaiiGacqWFdpWCdaqhaaWcbaGaeyOeI0cabaGaeyOeI0IaeGOmaidaaOGaeiiFaWhccmGae4NSdiMaeiOFa4Naem4raCKaemyyaeMaemyBa0MaemyBa0Maemyyae2aaeWaaeaadaWcaaqaaiqbdchaWzaaiaWaaSbaaSqaaiabgkHiTaqabaaakeaacqaIYaGmaaGaeiilaWYaaeWaaeaadaWcaaqaaiabigdaXaqaaiab=z8aMnaaBaaaleaacqGHsislaeqaaaaakiabgUcaRmaalaaabaGaeGymaedabaGaeGOmaidaamaaqahabaGae8NSdi2aa0baaSqaaiabdUgaRbqaaiabikdaYaaaaeaacqWGRbWAcqGH9aqpcqaIXaqmaeaacqWGWbaCa0GaeyyeIuoakiabdMeajjabcUfaBjab=j7aInaaBaaaleaacqWGRbWAaeqaaOGaeyipaWJaeGimaaJaeiyxa0facaGLOaGaayzkaaWaaWbaaSqabeaacqGHsislcqaIXaqmaaaakiaawIcacaGLPaaacqGGUaGlaaa@61A1@

The parameters were initialized on the basis of estimators from univariate approaches. After the initial burn-in period (5,000 in the following analysis), the next 5,000 iterations in the Markov chain were used for inference without thinning. Convergence of the algorithm was checked by the diagnostic tools in Cowles and Carlin [42].

### Profiling criterion

The significance of each centroid in model (1) is determined by one pair of parameters. They are, for the *j*-th centroid, the posterior probabilities *p*_*k*+ _= *P*(*β*_*k *_> 0|D
 MathType@MTEF@5@5@+=feaafiart1ev1aaatCvAUfKttLearuWrP9MDH5MBPbIqV92AaeXatLxBI9gBamrtHrhAL1wy0L2yHvtyaeHbnfgDOvwBHrxAJfwnaebbnrfifHhDYfgasaacH8akY=wiFfYdH8Gipec8Eeeu0xXdbba9frFj0=OqFfea0dXdd9vqai=hGuQ8kuc9pgc9s8qqaq=dirpe0xb9q8qiLsFr0=vr0=vr0dc8meaabaqaciaacaGaaeqabaWaaeGaeaaakeaaimaacqWFdepraaa@3827@) and *p*_*k*- _= *P*(*β*_*k *_< 0|D
 MathType@MTEF@5@5@+=feaafiart1ev1aaatCvAUfKttLearuWrP9MDH5MBPbIqV92AaeXatLxBI9gBamrtHrhAL1wy0L2yHvtyaeHbnfgDOvwBHrxAJfwnaebbnrfifHhDYfgasaacH8akY=wiFfYdH8Gipec8Eeeu0xXdbba9frFj0=OqFfea0dXdd9vqai=hGuQ8kuc9pgc9s8qqaq=dirpe0xb9q8qiLsFr0=vr0=vr0dc8meaabaqaciaacaGaaeqabaWaaeGaeaaakeaaimaacqWFdepraaa@3827@). Given data D
 MathType@MTEF@5@5@+=feaafiart1ev1aaatCvAUfKttLearuWrP9MDH5MBPbIqV92AaeXatLxBI9gBamrtHrhAL1wy0L2yHvtyaeHbnfgDOvwBHrxAJfwnaebbnrfifHhDYfgasaacH8akY=wiFfYdH8Gipec8Eeeu0xXdbba9frFj0=OqFfea0dXdd9vqai=hGuQ8kuc9pgc9s8qqaq=dirpe0xb9q8qiLsFr0=vr0=vr0dc8meaabaqaciaacaGaaeqabaWaaeGaeaaakeaaimaacqWFdepraaa@3827@, the marginal posterior distribution of *β*_*k *_is still a mixture of three components, i.e., being positive with probability *p*_*k*+ _= *E*[w˜
 MathType@MTEF@5@5@+=feaafiart1ev1aaatCvAUfKttLearuWrP9MDH5MBPbIqV92AaeXatLxBI9gBaebbnrfifHhDYfgasaacH8akY=wiFfYdH8Gipec8Eeeu0xXdbba9frFj0=OqFfea0dXdd9vqai=hGuQ8kuc9pgc9s8qqaq=dirpe0xb9q8qiLsFr0=vr0=vr0dc8meaabaqaciaacaGaaeqabaqabeGadaaakeaacuWG3bWDgaacaaaa@2E32@_*k*+_|D
 MathType@MTEF@5@5@+=feaafiart1ev1aaatCvAUfKttLearuWrP9MDH5MBPbIqV92AaeXatLxBI9gBamrtHrhAL1wy0L2yHvtyaeHbnfgDOvwBHrxAJfwnaebbnrfifHhDYfgasaacH8akY=wiFfYdH8Gipec8Eeeu0xXdbba9frFj0=OqFfea0dXdd9vqai=hGuQ8kuc9pgc9s8qqaq=dirpe0xb9q8qiLsFr0=vr0=vr0dc8meaabaqaciaacaGaaeqabaWaaeGaeaaakeaaimaacqWFdepraaa@3827@], being negative with probability *p*_*k*- _= *E*[w˜
 MathType@MTEF@5@5@+=feaafiart1ev1aaatCvAUfKttLearuWrP9MDH5MBPbIqV92AaeXatLxBI9gBaebbnrfifHhDYfgasaacH8akY=wiFfYdH8Gipec8Eeeu0xXdbba9frFj0=OqFfea0dXdd9vqai=hGuQ8kuc9pgc9s8qqaq=dirpe0xb9q8qiLsFr0=vr0=vr0dc8meaabaqaciaacaGaaeqabaqabeGadaaakeaacuWG3bWDgaacaaaa@2E32@_*k*-_|D
 MathType@MTEF@5@5@+=feaafiart1ev1aaatCvAUfKttLearuWrP9MDH5MBPbIqV92AaeXatLxBI9gBamrtHrhAL1wy0L2yHvtyaeHbnfgDOvwBHrxAJfwnaebbnrfifHhDYfgasaacH8akY=wiFfYdH8Gipec8Eeeu0xXdbba9frFj0=OqFfea0dXdd9vqai=hGuQ8kuc9pgc9s8qqaq=dirpe0xb9q8qiLsFr0=vr0=vr0dc8meaabaqaciaacaGaaeqabaWaaeGaeaaakeaaimaacqWFdepraaa@3827@], and having a point mass at zero with probability 1 - *p*_*k*+ _- *p*_*k*-_. The two parameters *p*_*k*+ _and *p*_*k*- _can be estimated from the Markov chains of w˜
 MathType@MTEF@5@5@+=feaafiart1ev1aaatCvAUfKttLearuWrP9MDH5MBPbIqV92AaeXatLxBI9gBaebbnrfifHhDYfgasaacH8akY=wiFfYdH8Gipec8Eeeu0xXdbba9frFj0=OqFfea0dXdd9vqai=hGuQ8kuc9pgc9s8qqaq=dirpe0xb9q8qiLsFr0=vr0=vr0dc8meaabaqaciaacaGaaeqabaqabeGadaaakeaacuWG3bWDgaacaaaa@2E32@_*βk*+ _and w˜
 MathType@MTEF@5@5@+=feaafiart1ev1aaatCvAUfKttLearuWrP9MDH5MBPbIqV92AaeXatLxBI9gBaebbnrfifHhDYfgasaacH8akY=wiFfYdH8Gipec8Eeeu0xXdbba9frFj0=OqFfea0dXdd9vqai=hGuQ8kuc9pgc9s8qqaq=dirpe0xb9q8qiLsFr0=vr0=vr0dc8meaabaqaciaacaGaaeqabaqabeGadaaakeaacuWG3bWDgaacaaaa@2E32@_*βk*- _drawn from the above Gibbs sampler. With moderately large *p*, the upper bound 2n
 MathType@MTEF@5@5@+=feaafiart1ev1aaatCvAUfKttLearuWrP9MDH5MBPbIqV92AaeXatLxBI9gBaebbnrfifHhDYfgasaacH8akY=wiFfYdH8Gipec8Eeeu0xXdbba9frFj0=OqFfea0dXdd9vqai=hGuQ8kuc9pgc9s8qqaq=dirpe0xb9q8qiLsFr0=vr0=vr0dc8meaabaqaciaacaGaaeqabaqabeGadaaakeaadaGcaaqaaiabd6gaUbWcbeaaaaa@2E2C@/*p *on (*w*_+ _+ *w*_-_) may not be restrictive and *β*_*k *_can be estimated with the median value of its posterior probability. However, *p *is usually much larger than *n *in gene expression data and, as a result, *p*_*k*+ _and *p*_*k*- _may be heavily shrunk to zero. Therefore, identifying significant prognostic centroids with the posterior median values will be too conservative. Instead, we suggest profiling the prognostic association of *k*-th centroid to the event time by

p˜
 MathType@MTEF@5@5@+=feaafiart1ev1aaatCvAUfKttLearuWrP9MDH5MBPbIqV92AaeXatLxBI9gBaebbnrfifHhDYfgasaacH8akY=wiFfYdH8Gipec8Eeeu0xXdbba9frFj0=OqFfea0dXdd9vqai=hGuQ8kuc9pgc9s8qqaq=dirpe0xb9q8qiLsFr0=vr0=vr0dc8meaabaqaciaacaGaaeqabaqabeGadaaakeaacuWGWbaCgaacaaaa@2E24@_*k *_= max{*p*_*k*+_, *p*_*k*-_},     (2)

which is a relative measure when *p *is much larger than *n*. As demonstrated in the simulation study, this profiling criterion performs much better than the popular univariate Cox score.

## Authors' contributions

DZ and MZ both contributed to the development of the modeling method. DZ wrote the Matlab code and did the simulation study. MZ analyzed the real data. Both authors read and approved the final manuscript.
